# A large genomic island allows *Neisseria meningitidis* to utilize propionic acid, with implications for colonization of the human nasopharynx

**DOI:** 10.1111/mmi.12664

**Published:** 2014-06-27

**Authors:** Maria Chiara E Catenazzi, Helen Jones, Iain Wallace, Jacqueline Clifton, James P J Chong, Matthew A Jackson, Sandy Macdonald, James Edwards, James W B Moir

**Affiliations:** Department of Biology, University of YorkHeslington, York, YO10 5DD, UK

## Abstract

*Neisseria meningitidis* is an important human pathogen that is capable of killing within hours of infection. Its normal habitat is the nasopharynx of adult humans. Here we identify a genomic island (the *prp* gene cluster) in *N. meningitidis* that enables this species to utilize propionic acid as a supplementary carbon source during growth, particularly under nutrient poor growth conditions. The *prp* gene cluster encodes enzymes for a methylcitrate cycle. Novel aspects of the methylcitrate cycle in *N. meningitidis* include a propionate kinase which was purified and characterized, and a putative propionate transporter. This genomic island is absent from the close relative of *N. meningitidis*, the commensal *Neisseria lactamica*, which chiefly colonizes infants not adults. We reason that the possession of the *prp* genes provides a metabolic advantage to *N. meningitidis* in the adult oral cavity, which is rich in propionic acid-generating bacteria. Data from classical microbiological and sequence-based microbiome studies provide several lines of supporting evidence that *N. meningitidis* colonization is correlated with propionic acid generating bacteria, with a strong correlation between *prp*-containing *Neisseria* and propionic acid generating bacteria from the genus *Porphyromonas*, and that this may explain adolescent/adult colonization by *N. meningitidis*.

## Introduction

Bacterial pathogens, such as *Neisseria meningitidis* (a.k.a. the meningococcus), are adapted to suit the host environment. In addition to the host–pathogen interaction, the microbe is part of a diverse and complex microbiota, in which it cooperates and competes for resources with its neighbours. *N. meningitidis* is notorious for its ability to cause severe sepsis and/or meningitis within a matter of hours following its acquisition and the movement of the bacterium into the bloodstream (van Deuren *et al*., 2000). However, disease is a rare outcome of *N. meningitidis* acquisition. Typically the bacterium lives harmlessly in the human nasopharynx of 5–40% of the population at any given time (Cartwright *et al*., 1987; Caugant *et al*., 1994; Sim *et al*., 2000; Yazdankhah and Caugant, 2004). *N. meningitidis* is most closely related to the sexually transmitted pathogen *Neisseria gonorrhoeae*, but other members of the *Neisseria* genus are non-pathogens. *Neisseria lactamica* is the non-pathogen that is most closely related to the pathogenic *Neisseria* species, and is of particular clinical relevance since it contributes to the development of immunity to meningococcal disease in childhood (Gold *et al*., 1978) and following experimental inoculation with *N. lactamica* in adults (Evans *et al*., 2011). *N. meningitidis* and *N. lactamica* both colonize the human nasopharynx as their natural habitat, but *N. lactamica* colonization is largely confined to infants, whereas *N. meningitidis* colonization increases with age from birth, reaching a peak in late teenage and declining in older adults (Gold *et al*., 1978; Cartwright *et al*., 1987; Olsen *et al*., 1991; Christensen *et al*., 2010). The incidence of meningococcal disease is highest among infants (with a naïve immune system) but a secondary age related peak in disease is seen among adolescents and young adults, which correlates with meningococcal carriage frequency (Jones and Mallard, 1993). Ultimately, the reservoir of adult meningococcal colonization is crucial for the occurrence of epidemic meningococcal disease (in both adults and children), and thus it is important to understand what enables *N. meningitidis*, but not *N. lactamica*, to effectively colonize adults.

**Fig 1:**
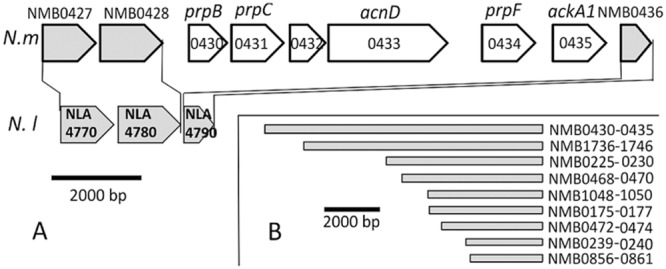
A. Comparison of genomic region containing and flanking NMB0430–0435 between *N. meningitidis* MC58, denoted *N.m*, and *N. lactamica* 020-06, denoted *N.l*. The genes NMB0430–0435 are shown in white (to scale), the flanking genes are shown in grey.B. Relative sizes of the multigene clusters found in *N. meningitidis* but absent from *N. lactamica*.

An analysis of the genomic differences between *N. lactamica* and the two pathogenic *Neisseria* species reveals nine genomic islands that are found in all *N. meningitidis* strains, but are absent from all four *N. lactamica* strains currently available in NCBI database (ST640, N5195, ATCC23970 and Y92-1009) (Fig. 1). The largest of these DNA regions is a 10200 base pair region which contains genes NMB0430–NMB0435 (of the serogroup B strain *N. meningitidis* MC58). This region is also conserved in all *N. gonorrhoeae* strains. Homologous DNA sequences flanking this region (NMB0428 and NMB0436) are adjacent to one another (separated by less than 100 base pairs) in *N. lactamica* (Fig. 1) and other closely related *Neisseria* species *N. polysaccharea* and *N. cinerea* indicating that the NMB0430–0435 gene cluster is a recent horizontally transferred acquisition. More distantly related *Neisseria* species (for a *Neisseria* genus phylogeny, see e.g. Marri *et al*., 2010), *N. subflava*, *N. flavescens*, *N. macaceae*, *N. sicca* and *N. mucosa* contain homologues of the genes from this island, bearing very high levels of identity (typically 97–99% identical), but the flanking genes are not the same as found in *N. meningitidis*. All this is consistent with the NMB0430–0435 gene cluster being a horizontally transferred acquisition either from more distantly related members of the *Neisseria* genus or from outside the genus.

**Fig 2:**
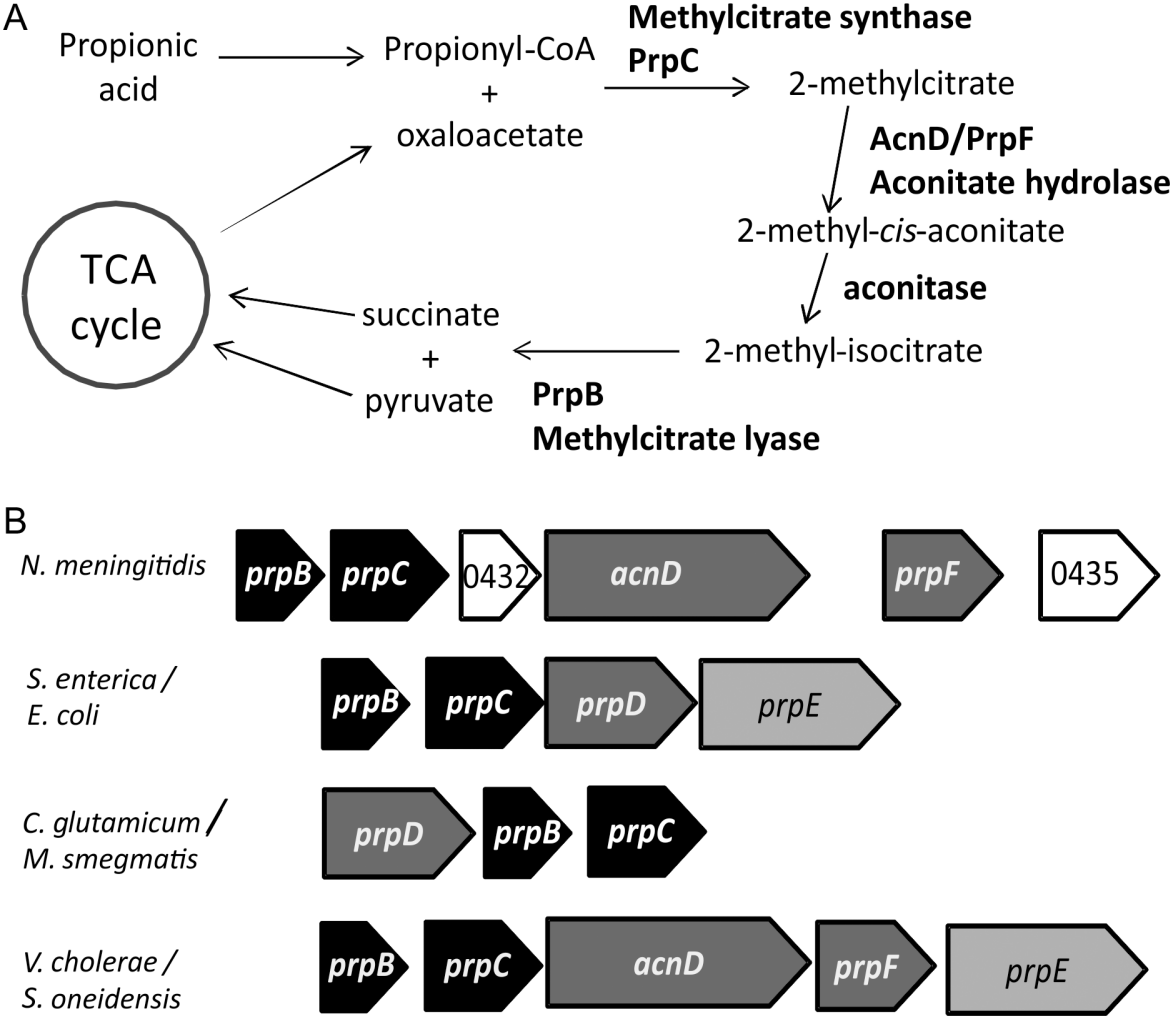
A. A scheme of the methylcitrate cycle, indicating the roles of genes from the NMB0430–0435 gene cluster, predicted based on their similarity to genes of established function in other bacteria.B. Comparison of NMB0430–0435 gene cluster with *prp* clusters from other bacteria. *prpB* and *prpC* homologues are shown in black, 2-methylcitrate dehydratases *acnD*/*prpF* and *prpD* are shown in dark grey. *prpE* is shown in pale grey. Genes of unknown function are shown in white.

Genes within the NMB0430–NMB0435 cluster have homology to genes involved in the methylcitrate cycle from other bacteria (Horswill and Escalante-Semerena, 2001). This pathway is used in other bacteria [e.g. *Salmonella enterica* (Hammelman *et al*., 1996; Horswill and Escalante-Semerena, 1999b), *Escherichia coli* (Textor *et al*., 1997; London *et al*., 1999), *Ralstonia eutropha* (Bramer and Steinbuchel, 2001), *Burkholderia sacchari* (Bramer *et al*., 2002), *Corynebacterium glutamicum* (Claes *et al*., 2002), *Mycobacterium tuberculosis* (Munoz-Elias *et al*., 2006) and *Mycobacterium smegmatis* (Upton and McKinney, 2007)] to convert propionic acid to pyruvate (Fig. 2A), which supports growth and/or limits the toxicity of propionic acid. NMB0430 and NMB0431 are homologues of methylisocitrate lyase (*prpB*) (Grimek *et al*., 2003) and methylcitrate synthase (*prpC*) (Horswill and Escalante-Semerena, 1999a) respectively. NMB0433 and NMB0434 are homologues of *acnD* and *prpF* from *Shewanella oneidensis* and *Vibrio cholerae* that catalyse methylcitrate dehydratase activity, acting as a functional replacement for the gene *prpD* that is used in the methylcitrate cycle in the enteric bacteria, *C. glutamicum* and *M. smegmatis* (Horswill and Escalante-Semerena, 1999a; Grimek and Escalante-Semerena, 2004) (Fig. 2B). Propionate activation is achieved by its conversion to propionyl CoA as the first step in the methyl citrate cycle in *S. enterica* via PrpE (Horswill and Escalante-Semerena, 1999a). However, no homologue of PrpE is found in *N. meningitidis*. The other genes in the NMB0430–NMB0435 gene cluster are NMB0432, which encodes a putative membrane protein, and NMB0435 which encodes a putative acetate kinase (*ackA1*).

Here we demonstrate for the first time that *N. meningitidis* can utilize propionic acid as a carbon source, and that this is dependent on possession of the NMB0430–0435 gene cluster. We propose that the ability of *N. meningitidis* to utilize propionic acid provides it with a selective advantage in the adult nasopharynx which is rich in anaerobes, many of which produce propionic acid as an end-product of fermentation.

## Results and discussion

### The *prp* gene cluster enables *N. meningitidis* to utilize propionic acid as a carbon source

**Fig 3:**
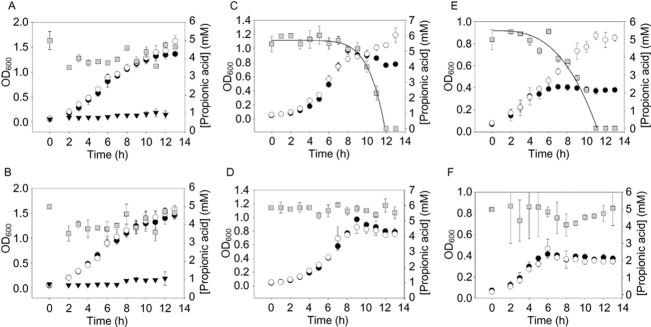
Growth and propionic acid content in *N. meningitidis*. Wild-type strain MC58 (panels A, C, and E), *prpC*-deficient strain NMB0431::spec^r^ (panels B, D and F). Cultures were grown in Mueller–Hinton broth (A and B), chemically defined medium with glucose (C and D) or pyruvate (E and F) as main carbon source. Growth was followed with no propionic acid (filled circles), 5 mM propionic acid (open circles) and 10 mM propionic acid (filled inverted triangles). Propionic acid content was followed for cultures inoculated with 5 mM propionic acid and is shown as grey squares. Data are means ± standard deviation for three independent experiments.

To test whether the NMB0430–NMB0435 gene cluster (henceforth referred to as the *prp* gene cluster) is functional in *N. meningitidis*, we introduced an antibiotic resistance cassette into NMB0431 (*prpC*, the putative methylcitrate synthase). *prpC* does not contribute to increased growth or propionic acid resistance in *N. meningitidis* grown in rich media (Fig. 3A and B). However, in chemically defined media with glucose or pyruvate as carbon source, propionic acid supplements growth of *N. meningitidis* (Fig. 3C and E). The extended growth in late exponential phase induced by propionic acid is concomitant with propionic acid depletion, and is dependent on *prpC* (Fig. 3C–F).

**Fig 4:**
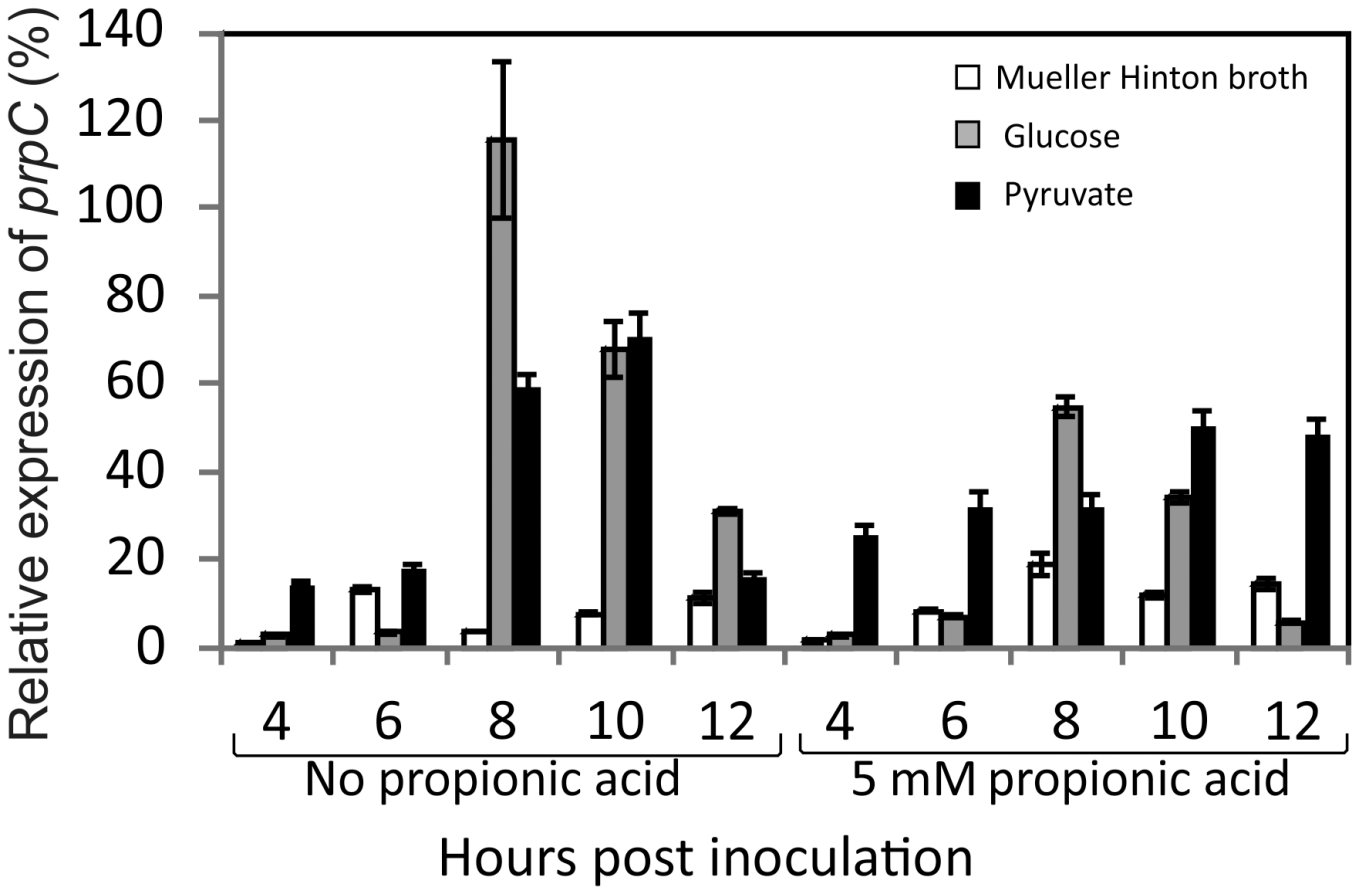
Relative expression of *prpC* over growth time-course in Mueller–Hinton broth (white bars), chemically defined medium with glucose as carbon source (grey bars) and chemically defined medium with pyruvate as carbon source (black bars). Expression was assessed using quantitative real-time PCR. Expression levels are shown for cultures ± 5 mM propionic acid. Data are means ± standard deviation for at least three independent experiments.

The expression of *prpC* is highly regulated during growth in different media (Fig. 4). Expression of *prpC* is considerably lower in Mueller–Hinton broth (rich media) than in chemically defined media with glucose or pyruvate as the major carbon source. *prp* gene expression is not induced directly by propionic acid (Fig. 4). Expression of *prpC* increases towards stationary phase in chemically defined medium with either glucose or pyruvate as carbon source, consistent with its induction being responsive to nutrient poverty. NMB0431 has previously been shown to be regulated by a global regulator of nutrient limitation (NMB0573; Lrp) in *N. meningitidis* (Ren *et al*., 2007). The *prp* gene cluster has also been shown to be regulated by a small RNA (Bsn1) (Del Tordello *et al*., 2012), although whether this regulation is related to nutrient deprivation and/or Lrp is not currently known.

Propionic acid utilization begins earlier in the growth of *N. meningitidis* in pyruvate medium, compared to glucose medium, corresponding with a high level of expression of *prpC* after 4 or 6 h of culturing, whereas propionic acid utilization in glucose media occurs rapidly, but only after around 8 h of incubation, consistent with a sharp increase in *prp* expression after this time (Figs 3 and 4). Overall, the transcriptional regulation of the *prp* gene cluster is sufficient to explain the different propionic acid utilization patterns in different media.

**Fig 5:**
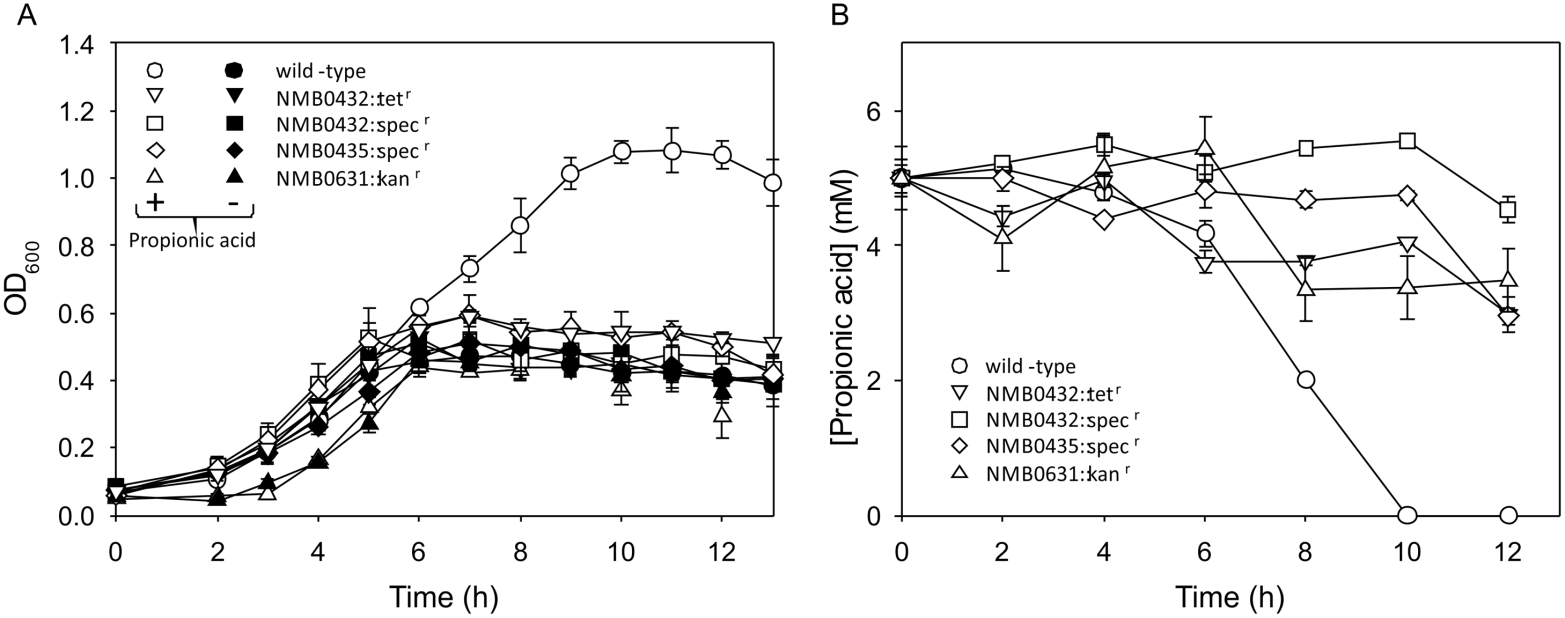
Growth (A) and propionic acid utilization (B) in *N. meningitidis* MC58 (circles), NMB0432::tet^r^ (inverted triangles), NMB0432::spec^r^ (squares), NMB0435::spec^r^ (diamonds) and NMB0631::kan^r^ (triangles). Filled symbols represent growth in chemically defined medium with pyruvate as major carbon source, and open symbols represent growth in pyruvate + 5 mM propionic acid. Data are means ± standard deviation for at least three independent experiments.

**Fig 6:**
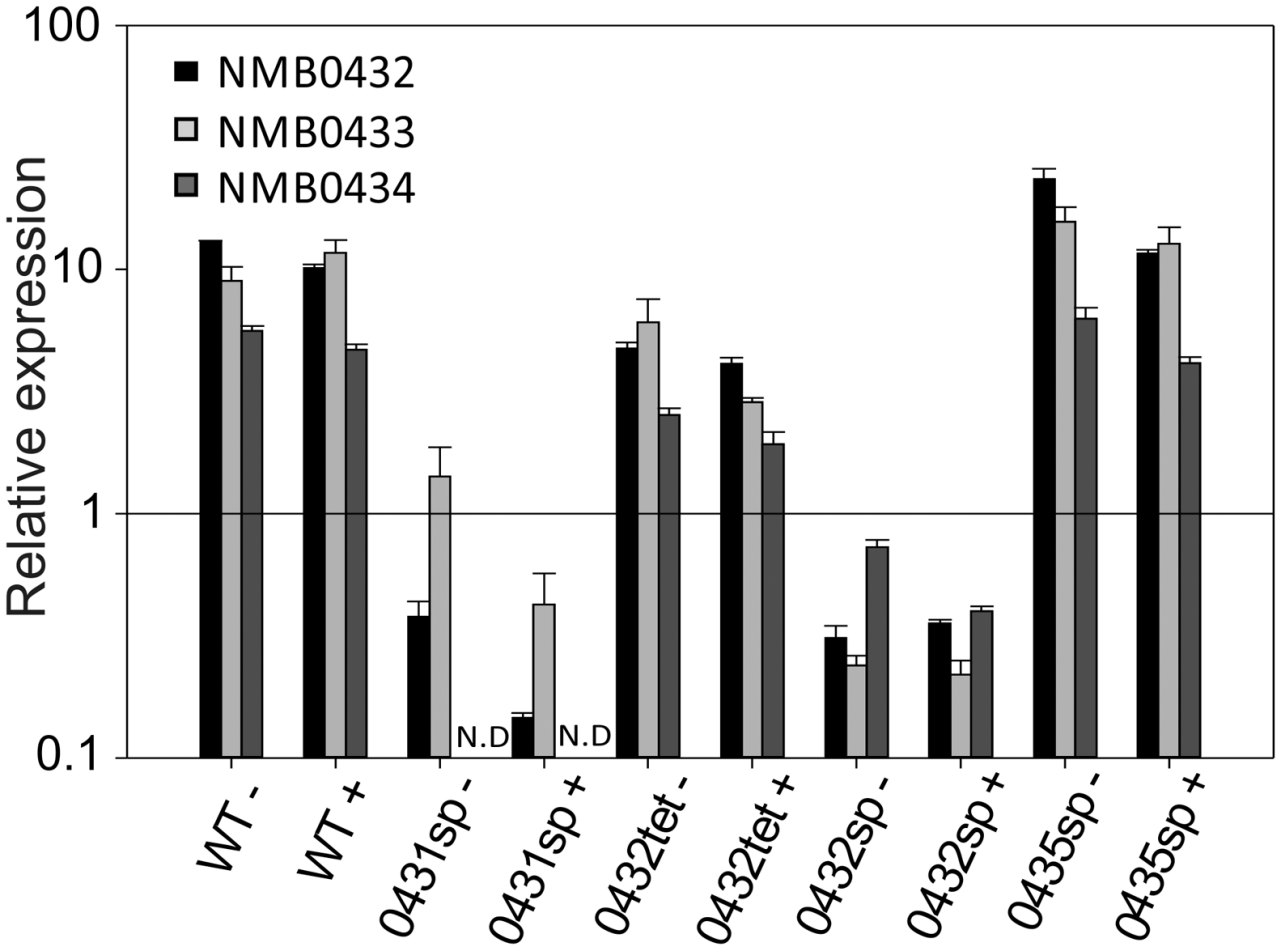
The relative expression of the NMB0432 (black bars), NMB0433 (pale grey bars) and NMB0434 (dark grey bars) in wild-type (WT), and mutant strains NMB0431::spec^r^ (0431sp), NMB0432::tet^r^ (0432tet), NMB0432::spec^r^ (0432sp) and NMB0435::spec^r^ (0435sp). Expression was assessed using quantitative real-time PCR. Expression was determined in cultures grown for 6 h in pyruvate medium with (denoted by +) or without (denoted by −) propionic acid. Data are expressed as relative expression compared to that of a wild-type culture grown for 6 h in MHB without propionic acid. N.D denotes not done. Error bars represent standard deviation and data are mean averages from at least three independent experiments.

Genes in the *prp* cluster NMB0430, NMB0433 and NMB0434 are highly similar to genes required for methylcitrate cycle action in other bacteria, but NMB0432 and NMB0435 are not similar to methylcitrate cycle genes from other organisms. To determine whether these genes are involved in propionic acid utilization in *N. meningitidis* we constructed gene knockouts in both these genes of unknown function. Mutants deficient in NMB0432 and NMB0435 were constructed by insertion of a spectinomycin resistance cassette into these open reading frames. Mutants deficient in NMB0432 or NMB0435 are unable to utilize propionic acid, and grow identically to the wild-type in the absence of propionic acid (Fig. 5) indicating that both these genes are required for propionic acid utilization in *N. meningitidis*. The spectinomycin resistance cassette used for generation of the knockout mutations contains powerful transcriptional terminators (Prentki and Krisch, 1984), and is anticipated to have an effect on transcription of genes downstream. Therefore, a mutant was also constructed in which NMB0432 was disrupted with a tetracycline resistance cassette lacking transcriptional terminators. Expression of genes NMB0433 and NMB0434, immediately downstream from NMB0432, was retained in this tetracycline-resistant mutant (albeit at lower levels than in wild-type or NMB0435-deficient strains), but was severely downregulated in the NMB0432::Spec^r^ construct (Fig. 6). This finding and further experimental evidence (Fig. S1) indicate that the *prp* genes are expressed in a single operon. Strain NMB0432::tet^r^ was unable to utilize propionic acid, and grew identically to the wild-type in the absence of propionic acid (Fig. 5) indicating that NMB0432 is required for propionic acid utilization in *N. meningitidis*.

### NMB0435 encodes a propionate kinase

The structure of the *prp* gene cluster in *N. meningitidis* is distinct from that of other bacteria that contain a methylcitrate cycle in two unique respects. First, the gene NMB0435, which is annotated as acetate kinase *ackA1*, is not present in other bacteria. *N. meningitidis* MC58 encodes two putative acetate kinases: *ackA1* (NMB0435) and *ackA2* (NMB1518). To explain the presence of the *ackA1* gene in the *prp* gene cluster and to explain the phenotype of the NMB0435 mutant (unable to utilize propionic acid) we hypothesized that *ackA1* may in fact encode a propionate kinase. *ackA2* is presumed to encode a genuine acetate kinase in *N. meningitidis*. To test this, we overexpressed NMB0435 and NMB1518 in *E. coli* and assayed their activities with acetate and propionate as substrates in kinase assays (Table 1; Fig. S2). NMB1518 has acetate kinase activity, but it was not possible to measure an affinity of this enzyme for propionate since the data did not approach saturation, indicating that this is the *bona fide* acetate kinase in *N. meningitidis*. NMB0435, however, is active with both acetate and propionate as substrates, indicating it is capable of fulfilling a role in activation of propionate into propionyl phosphate as part of the methyl citrate cycle in *N. meningitidis*. The K_M_ of NMB0435 for propionate (16 mM) is higher, but within an order of magnitude, of that from the only other characterized propionate kinase: TdcD from *Salmonella* (K_M_ = 2.3 mM) (Simanshu *et al*., 2005). Preparations of NMB0435 have a low activity *in vitro* compared to NMB1518 (Fig. S2) and other propionate kinase characterized previously (Simanshu *et al*., 2005). This probably relates to the large number of cysteine residues (eight) in NMB0435 and the high concentrations of reducing agent (5 mM DTT) required for purification of active NMB0435. Typically, propionate utilizing bacteria use propionyl-CoA synthase (PrpE) (Horswill and Escalante-Semerena, 1999a) to activate propionate and provide the substrate for methylcitrate synthase (PrpC). *N. meningitidis* generates propionyl phosphate via NMB0435, and we hypothesize that this is converted to propionyl-CoA via phosphotransacetylase (NMB0631; Pta). Indeed, NMB0631-deficient mutants are unable to utilize propionic acid as an additional carbon source during growth (Fig. 5).

**Table 1:** Affinities of heterologously expressed kinases NMB0435 and NMB1518 for acetate and propionate

Enzyme	K_M acetate_ ± SD (mM)	K_M propionate_ ± SD (mM)
NMB0435 (AckA1)	20.8 ± 4.6	16.3 ± 2.2
NMB1518 (AckA2)	25.7 ± 4.3	Not measureable

The second innovation in the *prp* cluster in *N. meningitidis* is the gene NMB0432, homologues of which are not found in the methylcitrate cycle gene clusters in other organisms. It encodes a predicted membrane protein with six transmembrane spans. Mutant strains deficient in NMB0432 are unable to utilize propionic acid (despite expressing the other genes of the *prp* gene cluster) suggesting NMB0432 has a role in transporting propionate into the cell (although it is not possible to exclude the possibility that the phenotype on propionic acid is due to altered expression of downstream genes NMB0433–5). Pfam analysis indicates NMB0432 contains a domain (Pfam01925) similar to that of anion (sulphite) transporter TauE. Predicted membrane spans 4, 5 and 6 of NMB0432 each contain positively charged arginine or lysine residues, consistent with a role for this membrane protein in anion transport. Propionyl-CoA synthase PrpE has a much higher affinity for propionate (K_M_ = 20 μM) (Horswill and Escalante-Semerena, 2002) than does propionate kinase AckA1 (K_M_ = 16 mM), which may explain why an active transport system is required for propionate in *N. meningitidis*, but not in other propionate utilizing bacteria that possess PrpE. The K_M_ for propionate of propionate kinase is higher than the concentration of propionate provided in the media (5 mM) and higher than the *in vivo* concentration of propionate in the oral cavity [up to 600 μM in saliva (Takeda *et al*., 2009); from 1 mM in healthy to 10 mM in diseased subgingival crevice (Niederman *et al*., 1997)].

### *prpC* is not required for survival in blood

NMB0431 was identified as a highly upregulated gene in response to culturing *N. meningitidis* in human blood (Echenique-Rivera *et al*., 2011). We investigated whether the possession of the *prp* cluster is important for survival in blood by culturing wild-type and NMB0431::spec^r^ with whole human blood. There was no significant difference in the survival rates between the two strains indicating that this pathway is not crucial for meningococcal survival in blood (Fig. S3).

### A role for propionic acid metabolism in *N. meningitidis* colonization

**Fig 7:**
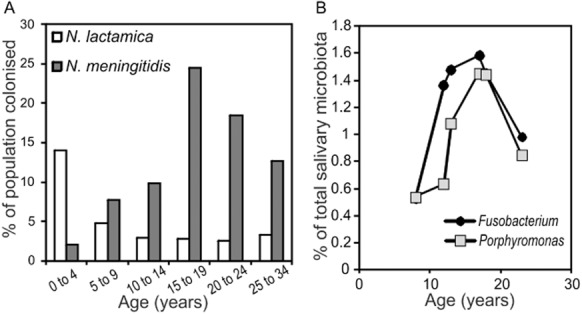
Similar age-dependent distribution in nasopharyngeal colonization of *N. meningitidis* (but not *N. lactamica*) compared to salivary content of genera *Porphyromonas* and *Fusobacterium*. *Neisseria* data (A) are from Cartwright *et al*. (1987); *Porphyromonas* and *Fusobacterium* data (B) are from Stahringer *et al*. (2012). The *Neisseria* data refer to the percentage of the population colonized by *N. lactamica* or *N. meningitidis* and are shown as bars based on age groupings 0–4, 5–9, 10–14, 15–19, 20–24 and 25–34. The *Porphyromonas* and *Fusobacterium* data refer to the percentage of total 16S rRNA reads that were identified as belonging to these genera among salivary samples from individuals aged 8, 12, 13, 17, 18 and 23.

The finding that the *prp* gene cluster possession does not affect survival in blood suggests that the function of this gene cluster may relate to survival in the normal habitat of *N. meningitidis* (i.e. the adult nasopharynx) rather than it having a role in virulence *per se*. While *N. meningitidis* is a common colonist of adults, *N. lactamica* is largely confined to infants (Gold *et al*., 1978; Cartwright *et al*., 1987; Olsen *et al*., 1991; Christensen *et al*., 2010). The finding that the methylcitrate cycle is not present in the infant-specific *N. lactamica* suggests that this metabolic pathway and propionate as a carbon/energy source might be important for enabling *N. meningitidis* to colonize older individuals. The main source of propionic acid in the oral cavity and naso-/oro-pharynx is via strict anaerobes that generate propionic acid as an end-product of fermentation. Anaerobes are acquired from birth along with aerobes, but the oral (Kononen *et al*., 1999; Kononen, 2005) and nasopharyngeal (Kononen *et al*., 2003) microflora of infants is dominated by aerobes, and the proportion of anaerobes increases with age through childhood into adolescence. Propionibacteria (e.g. *Propionibacterium acnes*) are an important source of propionic acid (generated as a product of sugar fermentation) on the human body. The number of propionic acid bacteria colonizing the nares (nostrils) changes significantly with age, increasing *c*. 100-fold from 6 years to 13 years (Mourelatos *et al*., 2007). Recent culture-independent studies of microbial colonization of the nasopharynx and upper respiratory tract provide further evidence in support of a change in the microflora between infants and adults. A study of 96 infants (18 months old) revealed the dominant bacterial colonists of the nasopharynx are the aerobic and facultatively anaerobic genera *Moraxella* (40%) and *Haemophilus* (20%) respectively (Bogaert *et al*., 2011). The adult nasopharynx has a more complex microbiota and is dominated by Firmicutes, Proteobacteria, Bacteroidetes and Fusobacteria (Charlson *et al*., 2010). *Neisseria* is more common in adults and clusters with colonization by anaerobes of the genera *Porphyromonas* and *Fusobacterium* (Charlson *et al*., 2010) both of which produce propionic acid as end-products of amino acid fermentation (Carlier *et al*., 1997; Takahashi *et al*., 2000). Notably, the proportion of the salivary microbiota belonging to genera *Porphyromonas* and *Fusobacterium* varies with age, such that there is a peak of colonization around age 18, correlating tightly with changes in nasopharyngeal meningococcal carriage with age (our analysis of data in Stahringer *et al*., 2012; Fig. 7). Positive correlations were observed between *Neisseria* and *Porphyromonas* colonization between individuals in the oral cavity in two separate studies of diversity in the upper respiratory tract involving 91 individuals (Human Microbiome Project, accession SRX055077) and 293 individuals (Charlson *et al*., 2010) and two separate studies of bacterial diversity in saliva involving 120 individuals (Nasidze *et al*., 2009) and 278 individuals (Stahringer *et al*., 2012) (Supplementary Fig. S4 and Datasets S1–S6). *Porphyromonas* was the only genus consistently correlated with *Neisseria* among all four studies. For the throat samples it was possible to identify *Neisseria* to species level, with the dominant species being *N. subflava* (which contains the *prp* gene cluster) and *N. cinerea* (which lacks the *prp* gene cluster). *N. subflava* is positively correlated with *Porphyromonas* [Pearson correlations of 0.7215 and 0.3678 for the datasets from SRX055077 and (Charlson *et al*., 2010) respectively], whereas there is no correlation between *N. cinerea* and *Porphyromonas* (Pearson correlations of −0.0677 and −0.0363). Thus, the evidence from analysis of microbial communities indicates that the adult upper respiratory tract is enriched in propionic acid generating bacteria, providing a selection pressure for retention of a horizontally acquired metabolic pathway that allows utilization of propionate, as found in *N. meningitidis*.

### Conclusion

In this article we provide the first report that *N. meningitidis* contains a functioning methylcitrate cycle and is able to utilize the enzymes in this cycle to catalyse the breakdown of propionate. The utilization of propionate helps support growth of *N. meningitidis*, particularly under conditions of nutrient limitation. The genes encoding the methylcitrate cycle are located on the largest genomic island in *N. meningitidis*, an island that is present in the other pathogenic *Neisseria* (*N. gonorrhoeae*) but absent from closely related non-pathogenic *Neisseria* species *N. lactamica*, *N. cinerea* and *N. polysaccharea*.

Genomic islands in pathogens are typically referred to as pathogenicity islands. In this case, it appears that the *prp* gene cluster is not a pathogenicity island *per se*, but confers a metabolic capacity relevant to the natural habitat of the meningococcus. That the *prp* gene cluster is not central to virulence is in keeping with the fact that it appears to be universally maintained in *N. meningitidis* genomes, not just the hypervirulent lineages associated with most human disease. As such, the metabolic capacity to use propionic acid may be crucial in underpinning colonization of the *N. meningitidis* natural habitat (human adult upper respiratory tract), distinguishing it from the *N. lactamica* habitat (human infant upper respiratory tract). The capacity of *N. meningitidis* to colonize adolescents/adults is important for its transmission and disease epidemiology (Christensen *et al*., 2013). This increase in carriage in young adulthood is frequently attributed to increased social interaction and contact in this age group (see e.g. Christensen *et al*., 2010). While this is no doubt true, here we present for the first time a mechanistic explanation for why *N. meningitidis* in particular has increased carriage with age, based on the genetic properties of the meningococcus and co-colonizing microbiota in the host.

## Experimental procedures

### Strains, media and growth conditions

*Neisseria meningitidis* strain MC58 was used as wild-type control and as source of genomic DNA for mutant strain constructs. Its complete genome has been sequenced, published and annotated (Tettelin *et al*., 2000). *N. meningitidis* strains NMB0431::Spec^r^, NMB0432::Spec^r^, NMB0432::Tet^r^ and NMB0435::Spec^r^ were generated by allelic exchange of an interposon disrupted version of the gene with the wild-type, using similar methods to those described previously (Anjum *et al*., 2002). Genes to be mutated were amplified by PCR. NMB0431 was amplified with primers 5′-CCAAGCTTGTGTCGAAGCC and 5′-TTTCAGACGGCCTTTCCAATAAGG. NMB0432 was amplified with primers 5′-TTCTCTGCCGTTTCCTACCAA and 5′-ATGTCGGTTCTCCTGTGGAT. NMB0435 was amplified with primers 5′-TTGACGTAGCATGGGTTTGC and 5′-ACGCCCGAAATTCAAAATCC. In each case, the resultant product was cloned into pCR®-Blunt II-TOPO® vector (Invitrogen). The resultant plasmids were digested with PsiI (NMB0431), ClaI and AclI (NMB0432) and ClaI and BsmI (NMB0435) prior to insertion of the spectinomycin resistance gene from pHP45Ω (Prentki and Krisch, 1984) or the tetracycline resistance gene from pCMT18 (Heurlier *et al*., 2008). Resulting plasmids were transformed into *N. meningitidis* MC58 as described previously (Heurlier *et al*., 2008). *N. meningitidis* deficient in phosphotransacetylase (*pta*; NMB0631) was a kind gift from Dr Jon Shaw, University of Sheffield.

*Neisseria meningitidis* strains were streaked on Columbia Blood Agar Base plates (CBA) and incubated at 37°C overnight in a 5% CO_2_ atmosphere (Heurlier *et al*., 2008). Liquid cultures of *N. meningitidis* were grown in Mueller–Hinton broth (MHB) (Oxoid) or in chemically defined medium (CDM) modified from the method described by Catlin (Catlin and Schloer, 1962). CDM was prepared as described in Table S1. Both media where supplemented with 10 mM NaHCO_3_ prior to incubation. Liquid cultures were routinely grown from a starting OD_600_ reading of 0.05. Cultures were grown in triplicate in 30 ml polystyrene universal tubes (Sterilin®) in a total of 15 ml, and were shaken at 200 r.p.m. at 37°C in a microbial C25KC incubator shaker (New Brunswick Scientific). Growth in the presence of propionic acid was enabled by the addition of propionic acid (Sigma-Aldrich®) to a final concentration of 5 mM. Antibiotics were included as appropriate: spectinomycin, 50 μg ml^−1^; tetracycline 2.5 μg ml^−1^.

### Growth of *N. meningitidis* in whole blood

Human venous blood was collected from seven healthy adult volunteers (three males and four females) and an anti-coagulant agent, heparin, was instantly mixed in the blood at a concentration of 17 U ml^−1^. Samples were obtained from Wayne Burrill, University of Bradford, the morning subsequent to collection. *N. meningitidis* MC58 and NMB0431*::Spec^r^* were grown on plates overnight. A few colonies for each strain were inoculated the following day into MHB + 10 mM NaHCO_3_ until they reached an OD_600_ of 0.3, which corresponded to approximately 3 × 10^8^ bacteria·ml^−1^. Bacteria were subsequently diluted to 1 × 10^6^ or 2 × 10^6^ cfu ml^−1^ into MHB + 10 mM NaHCO_3_ and 10 μl of these suspensions were inoculated in triplicate into 190 μl of 100% human whole blood, resulting in a starting experimental concentration of between 5 × 10^4^ and 1 × 10^5^ cfu ml^−1^. Whole blood infected with bacteria was then incubated over a period of 2 h in a 96-Well Optical Reaction Plate (Applied Biosystems) at 37°C with shaking at 110 r.p.m. to avoid red blood cell sedimentation. At each time point (0, 30, 60, 90 and 120 min), 20 μl of sample was removed and spread onto CBA plates. The number of viable bacteria was determined by cfu counts by plating serial dilutions onto CBA plates. Bacterial survival was determined by comparison of the viable count at the different time points with the control time 0, where survival rate corresponded to 100%.

### Measurement of propionic acid content

Gas chromatography was used for separating propionic acid from growth medium. Five hundred microlitres of samples of culture were collected at different time points, centrifuged at 12 000 *g* for 5 min with a Sigma 1-13 microcentrifuge and the supernatant was stored at −80°C until the last sample was collected. Each sample to be analysed was then mixed with 132 mM potassium phosphate (pH 3). Half microlitre of freshly acidified samples were drawn up into a syringe, placed into a hot injector port of the 6890 N Network GC system gas chromatograph (Agilent Technologies), and the sample was injected. The injector port was at 250°C. Helium carrier gas flowed through the column (Alltech® AT-1000 Capillary Column; 150°C) at a constant flow of 2.2 ml min^−1^. The area under the curve of the propionic acid elution peak was measured and converted to the actual concentration of propionic acid with the help of a standard curve.

### Measurement of gene expression by RT-PCR

Messenger RNA and cDNA were prepared and the relative expression of genes determined as described previously (Heurlier *et al*., 2008) at time points throughout growth. Expression of NMB0431, NMB0432, NMB0433 and NMB0434 were determined relative to MetK (NMB1799) as endogenous control. Primers for RT-PCR are in Table S2.

To determine whether the *prp* gene cluster is expressed as a polycistronic operon, primers were designed to amplify across all the intergenic regions within the *prp* gene cluster and between NMB0428 and NMB0430, and NMB0435 and NMB0436 (i.e. the genes flanking the *prp* cluster). The primers are indicated in Fig. S1A, and shown in Table S2.

### Overexpression and purification of NMB0435 and NMB1518

*Neisseria meningitidis* MC58 NMB1518 (AckA2 acetate kinase) and NMB0435 (AckA1 suspected propionate kinase) genes were amplified by PCR with primers NMB1518F (5′-CCAGGGACCAGCAATGTCCCAAAAATTGATCTTGGTTTTG) and NMB1518R (5′-GAGGAGAAGGCGCGTTATTACAGACCGCTCAAACGGGCAGTGTCGTG) and NMB0435F (5′-CCAGGGACCAGCAATGTCCGACCAACTCATCCTCGTTCTGAAC) and NMB0435R (5′-GAGGAGAAGGCGCGTTACTACAAGATGCCGGCAAGTTCGGCAGTGT), respectively, and cloned into pETYSBLIC3C vectors (N-terminal His-tagged, *Kan^r^*) and transformed into *E. coli* BL21 (λ DE3), as described previously (Bonsor *et al*., 2006). Proteins were overexpressed by culturing in 650 ml autoinduction medium overnight at 30°C (Edwards *et al*., 2010). After induction, AckA2 cultures were pelleted at 4000 *g* for 15 min in a Sorvall Evolution RC centrifuge with an SLC-1500 rotor. Pellets were re-suspended in 30 ml nickel column equilibration buffer (50 mM Tris pH 7, 300 mM NaCl and 40 mM Imiadazole) and stored at −20°C until ready for purification. AckA1 cultures were treated with the same buffers, with the exception that all storage and purification buffers contained 5 mM DTT (Dithiothreitol) as a reducing agent. Purification and storage of AckA1 without at least 5 mM DTT yielded inactive protein. Pellets stored at −20°C were defrosted in 37°C water baths before sonication lysis with a MISONIX sonicator model 3000 for 3 min with 3 s on 7 s off pulses at 120 W. Total pellet lysate was then centrifuged at 10 000 *g* for 30 min in a Sigma 3-18 centrifuge with a Sigma 19776-H rotor to pellet insoluble cell debris. Crude cell lysate was then applied to 5 ml His-trap nickel columns previously equilibrated with equilibration buffer equivalent to five column volumes. After lysate had been applied, columns were then rinsed with another five column volumes of equilibration buffer before five column volumes of elution buffer [50 mM Tris pH 7, 300 mM NaCl, 500 mM Imidazole (+5 mM DTT for AckA1)] were applied; fractions equivalent to one column volume were collected from this. Purity was checked by SDS-PAGE on completion of the purification protocol. Acetate and propionate kinase activity was assayed using the hydroxamate assay as described previously (Aceti and Ferry, 1988), at a range of concentrations of substrate.

### Taxonomic analysis of 16S rRNA oral microbiome data

16S rRNA amplicon sequence data were downloaded from the NCBI Sequence Read Archive (http://www.ncbi.nlm.nih.gov/sra/) from four separate oral microbiome studies (Nasidze *et al*., 2009; Charlson *et al*., 2010; Stahringer *et al*., 2012; Human Microbiome Project, accession SRX055077). Adaptor sequences were trimmed using CutAdapt version 1.2.1 with an error rate of 0.2 and minimum length after trimming of 20 base pairs. QIIME version 1.7.0 was used to assign OTUs (operational taxonomic units) to the amplicon datasets, and run at both 97% and 99% similarity. The Greengenes (May 2013 version) was used to pick OTUs (with the UCLUST clustering algorithm) and assign taxa to representative sequences for each OTU. Relative abundance data produced by QIIME were used to calculate Pearson product moment correlations, using the SciPy stats module, between the taxa across the samples in each study, with correlation coefficients of ≤ −0.1175 or ≥ 0.1175 for the data from Stahringer *et al*. (2012), ≤ −0.1170 or ≥ 0.1170 for the data from Charlson *et al*., 2010), ≤ −0.18 or ≥ 0.18 for the data from Nasidze *et al*., 2009) and ≤ −0.3 or ≥ 0.3 for the data from SRX055077 being considered as significantly correlated.
