# Supplementary material for: A large genomic island allows Neisseria meningitidis to utilize propionic acid, with implications for colonization of the human nasopharynx
Source: Mol Microbiol. 2014 Jun 27;93(2):346–55. doi: 10.1111/mmi.12664 (PMC4441257; doi:10.1111/mmi.12664)
Supplement: Supplementary file 7 — Supporting Information [file mmi0093-0346-sd7.pdf]

Supplementary information

Fig. S1. The prp genes are expressed as an operon.

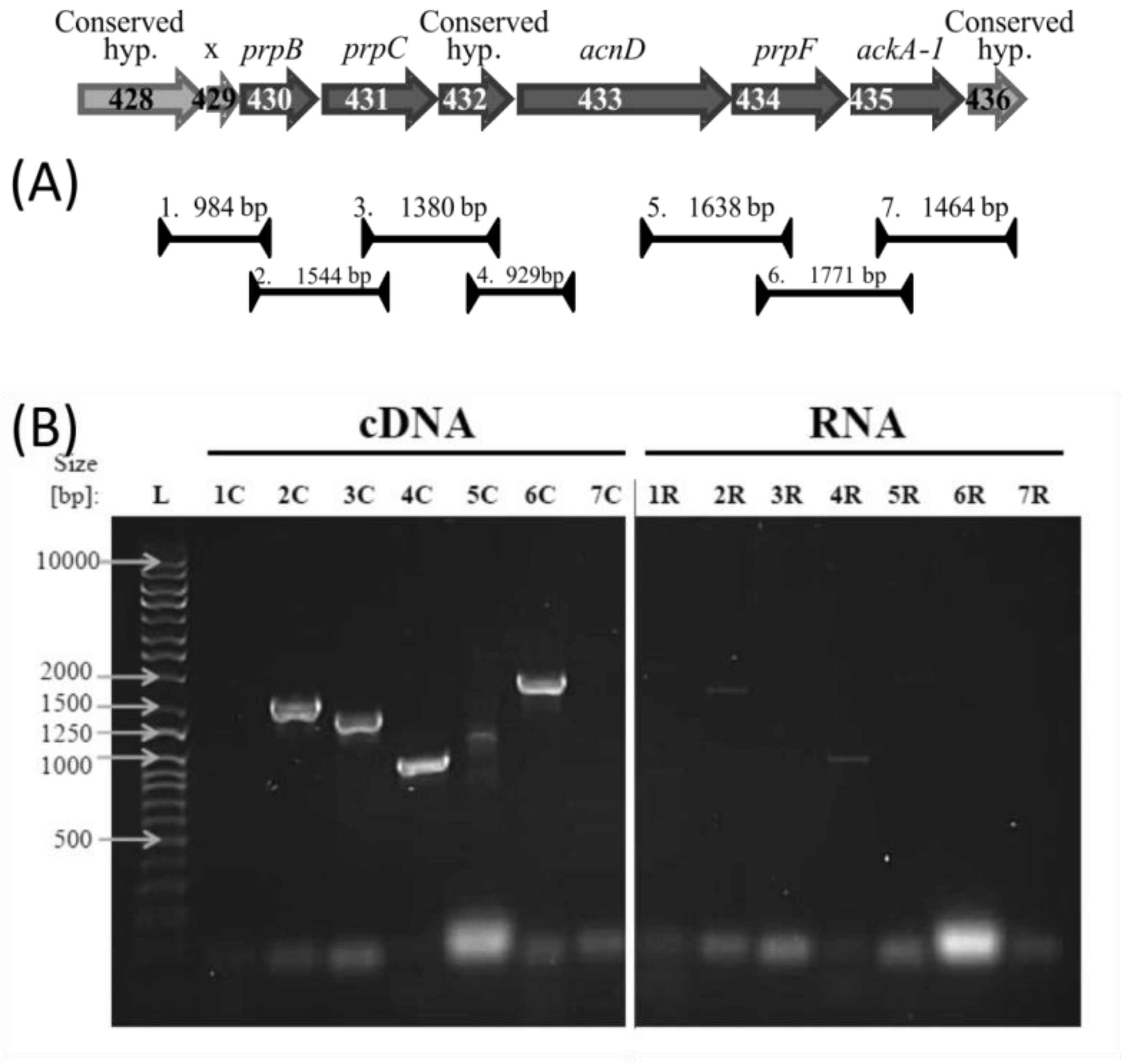

The observation that expression of downstream genes from the *prp* gene cluster are severely inhibited in NMB0432::spec^r and NMB0431::spec^r mutants (Fig. 6) indicates that these genes are in an operon in *N. meningitidis*. To obtain further evidence on this, we isolated mRNA and generated cDNA from cultures grown under conditions that lead to high expression of *prpC* (i.e. in chemically defined medium with pyruvate). Primer pairs were

designed from within each open reading frame in the *prp* gene cluster (and within flanking genes NMB0428 and NMB0436), in order to amplify across the intergenic regions using the cDNA as a template. Products were amplified for each pair of genes within the *prp* gene cluster but not between the *prp* gene cluster and the flanking genes on either side. Negative controls with mRNA that had not been reverse transcribed into cDNA showed that the products were not due to contamination with genomic DNA and indicated that the genes NMB0430-NMB0435 are co-transcribed in an operon.

Amplification of the intergenic regions of the prp cluster was determined using cDNA as a template, and mRNA as negative control. Wild-type bacteria were cultured in pyruvate + propionic acid for six hours to optimise *prp* cluster gene expression. Intergenic regions were amplified with primers specific to each of the open reading frames within the *prp* cluster and the flanking genes (NMB0428 and NMB0436) outside the prp cluster (panel A). The products were separated on 0.8 % agarose gels (B). The running order and expected sizes of products are as follows: lane 1: NMB0428-NMB0430, 984 bp (no fragment expected); lane 2: NMB0430-NMB0431, 1544 bp; lane 3: NMB0431-NMB0432, 1380 bp; lane 4: NMB0432-NMB0433, 929 bp; lane 5: NMB0433-NMB0434, 1638 bp; lane 6: NMB0434-NMB0435, 1771 bp; lane 7: NMB0435-NMB0436, 1464 bp (no fragment expected). Q-Step 4 Quantitative DNA ladder (YORBIO) (Lane L) was loaded on the gel to determine the size of the DNA bands seen.

Fig. S2. Purification and kinetic characterisation of AckA1 and AckA2

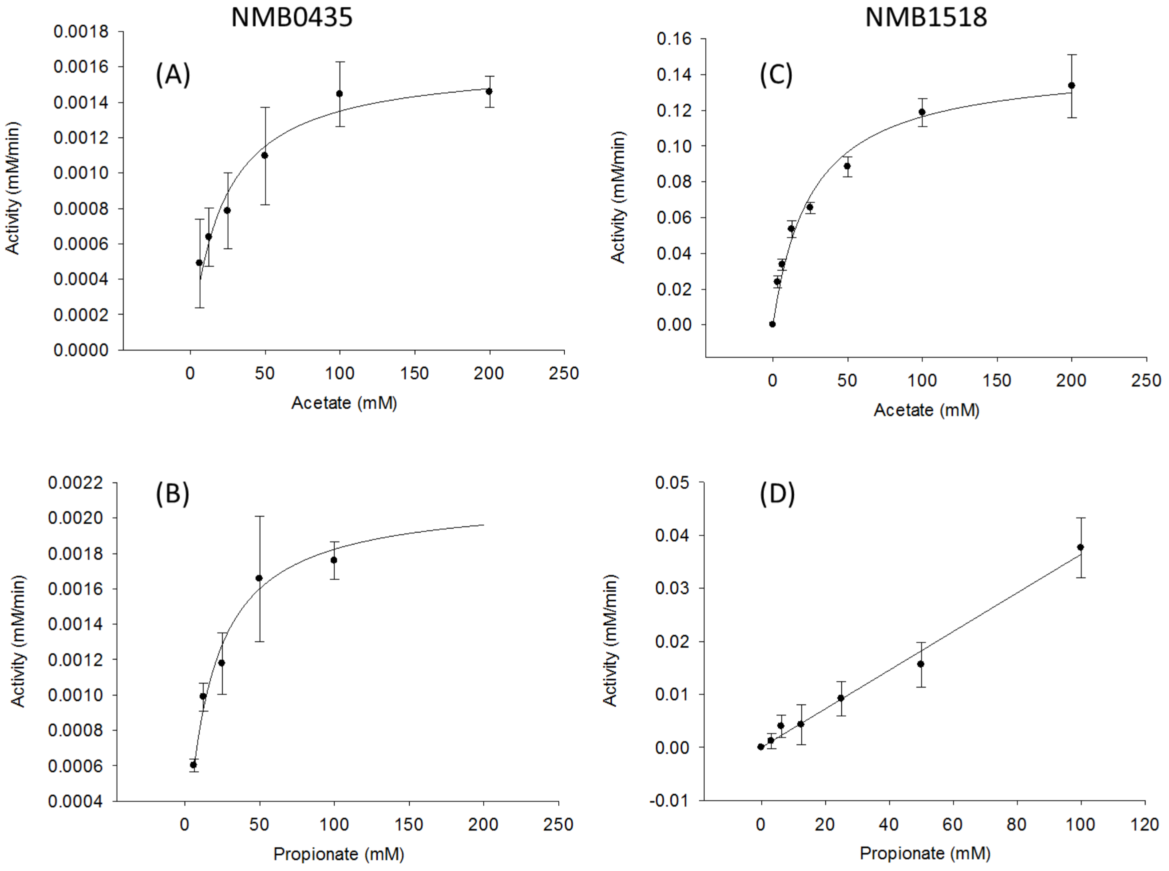

Affinity of AckA1/ NMB0435 for acetate (A) and propionate (B). Activity was measured with 2 µM purified enzyme. Affinity of AckA2/ NMB1518 for acetate (C) and propionate (D). The calculated K_M values are 20.7 mM (acetate) and 16.3 mM (propionate) for NMB0435 and 25.7 mM (acetate) and unmeasureable (propionate) for NMB1518. The activity of NMB0435 is considerably lower than NMB1518, which is probably related to the requirement for a high concentration of reducing agent (DTT) and the large number of cysteine residues (eight) in the NMB0435 protein sequence.

Fig. S3. Survival of *N. meningitidis* NMB0431::spec^r compared to wild-type in whole human blood.

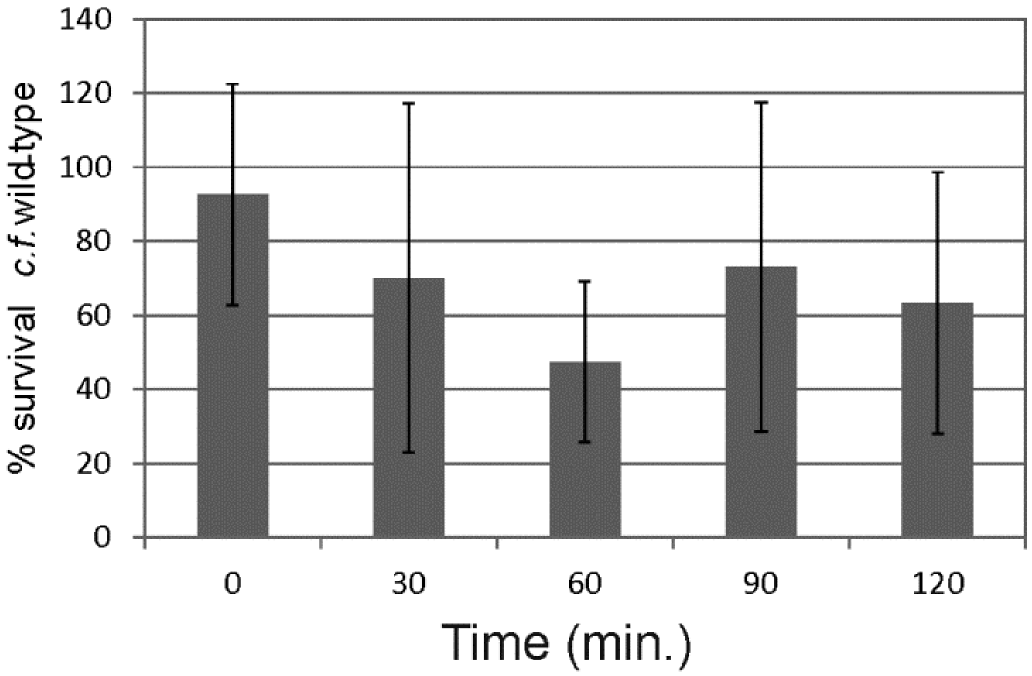

Cell cultures of *N. meningitidis* MC58 and NMB0431::spec^r were diluted into fresh human blood and cultured in 200 µl samples for 0, 30, 60, 90 and 120 minutes. The number of colony forming units was determined and the relative survival of the *prpC* mutant compared to the wild-type was determined in triplicate, at five time points, for six independent blood samples. There was no significant difference in survival between wild-type and *prpC* mutant over the time-course (Student's *t*-test, $p > 0.05$ for all samples).

Fig. S4. Correlations between Neisseria and other genera in human saliva and throat swab samples

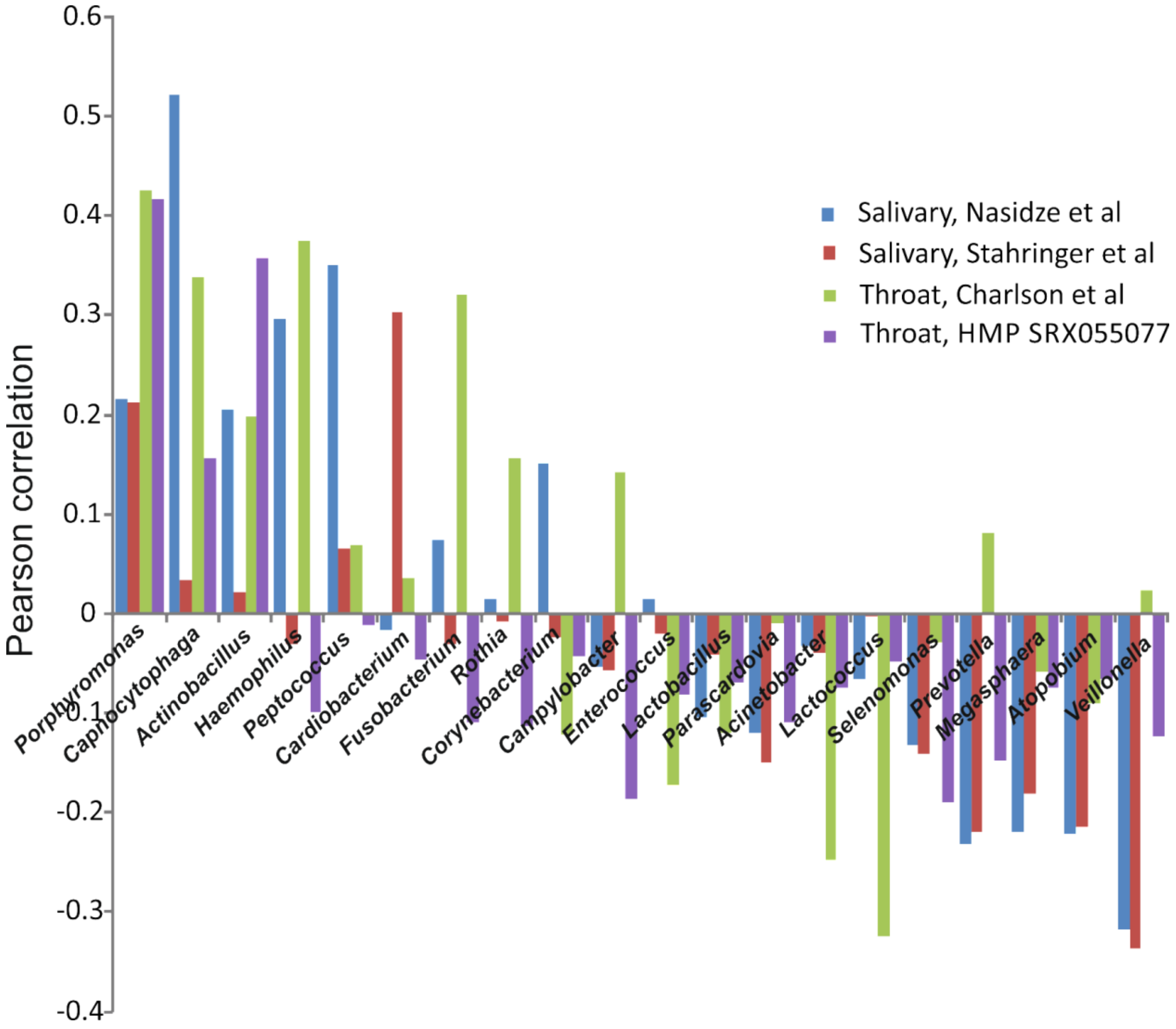

Pairwise correlations between colonisation with bacteria identified as operational taxonomic units (OTUs) at 97 % identity were assessed using Pearson's Product Moment Correlation for four different studies of 16S rRNA-based bacterial diversity [Nasidze et al., 2009, Stahringer et al., 2012, Charlson et al., 2010, and an unpublished study by the Human Microbiome Project, available on the Sequence Read Archive under accession number SRX055077]. Presented are correlations for genera identified as present in all four studies,

and in which there is a significant negative or positive correlation between *Neisseria* and the genus in question in at least one of the studies. The only genus which is consistently correlated with *Neisseria* in all four studies is *Porphyromonas*.

Supplementary Table S1. Constitution of Chemically defined media (CDM) used for growth of *N. meningitidis* strains.

CDM	Chemicals used	Stock concentration	Final concentration
**Solution 1 (Fe sol.) (40x)**	$MgCl_2$ (Sigma-Aldrich®)	78 mM	1.95 mM
	$CaCl_2$ (Sigma-Aldrich®)	8.15 mM	0.20 mM
	Ferric citrate (Sigma-Aldrich®)	6.5 mM	0.15 mM
	Dissolved in deionised H_2O, stirred at 50 °C for 3h approx., pH adjusted to 7 and filter sterilised		
**Solution 2 (salts sol.) (20x)**	NaCl (Sigma-Aldrich®)	2 M	100 mM
	K_2SO_4 (Sigma-Aldrich®)	114.8 mM	5.75 mM
	K_2HPO_4 (Sigma-Aldrich®)	460 mM	23 mM
	NH_4Cl (Sigma-Aldrich®)	360 mM	18 mM
	Dissolved in deionised H_2O, stirred at room temperature for 10 minutes prior to autoclaving		
**Solution 3 (aa sol.) (20x)**	Glycine (Sigma-Aldrich®)	75.6 mM	3.8 mM
	L-Cystine (Sigma-Aldrich®)	8.3 mM	0.4 mM
	L-Arginine (Sigma-Aldrich®)	14 mM	0.7 mM
	L-Glutamine (Sigma-Aldrich®)	80 mM	4 mM
	L-Serine (Sigma-Aldrich®)	95 mM	4.75 mM
	Dissolved in deionised H_2O with the addition of a few drops of NaOH, stirred at 40 °C for 1 hour and filter sterilised		
**Solution 4a* (224x)**	Glucose (Sigma-Aldrich®)	560 mM	2.5 mM
	Dissolved in deionised H_2O, stirred at room temperature for 10 minutes prior to autoclaving		
**Solution 4b* (40x)**	Na pyruvate (Sigma-Aldrich®)	200 mM	5 mM
	Stirred at room temperature for 30 minutes and filter sterilised		
**Solution 5 (100x)**	$NaHCO_3$ (Sigma-Aldrich®)	1 M	10 mM
	Dissolved in deionised H_2O, vortexed until complete dissolution of sodium		

	bicarbonate and filter sterilised		
**Solution 6 (200x)**	Propionic acid (Sigma-Aldrich®)	1 M	5 mM
	Dissolved in deionised H_2O, then adjusted to pH 7 prior to filter sterilisation		

*CDM media contained either glucose (solution 4a) or pyruvate (solution 4b).

Supplementary Table S2.

Primer name	Sequence (5' → 3')	Comment
For amplifying intergenic regions in and around prp gene cluster		
RT-NMB0428-for*	GCCCGCCCTGCTTTATGT	To amplify product 1, between NMB0428 and NMB0430
RT-NMB0430-rev*	TGGCCAATCGTGCAAAATAA	
RT-NMB0430-for*	GCCGTGAAAGAATCGAATCC	To amplify product 2, between NMB0430 and NMB0431
RT-NMB0431-rev	CGCGGGCGGTAAAGGTA	
RT-NMB0431-for	GCCATGCACGTTTCACTGAT	To amplify product 3, between NMB0431 and NMB0432
RT-NMB0432-rev	CCATCGTTGCCGCAATC	
RT-NMB0432-for	TGCAATCTTGGTTCGCTATCG	To amplify product 4, between NMB0432 and NMB0433
RT-NMB0433-rev	TGAGTCAGTACCGACGCAGGTA	
NMB0433b-for*	AGGCTTCGAGCGTATCCAC	To amplify product 5, between NMB0433 and NMB0434
RT-NMB0434-rev	CGTCGGCTGGATCAAGAAAT	
RT-NMB0434-for	AGCTCGACGGCGTAACGT	To amplify product 6, between NMB0434 and NMB0435
RT-NMB0435-rev*	GACGGATTTGCCGTTTTTGA	
RT-NMB0435-for*	CGCATGATTATTGCCCACTTAG	To amplify product 7, between NMB0435 and NMB0436
RT-NMB0436-rev*	TCGACGGCGTGTTCCAA	
For analysis of relative expression of *prp* genes compared to *metK* control.		
RT-NMB0431-for	GCCATGCACGTTTCACTGAT	RT-PCR of NMB0431
RT-NMB0431-rev	CGCGGGCGGTAAAGGTA	
RT-NMB0432-for	TGCAATCTTGGTTCGCTATCG	RT-PCR of NMB0432
RT-NMB0432-rev	CCATCGTTGCCGCAATC	
RT-NMB0433-for	CGCCCGTCGTCCAAGTC	RT-PCR of NMB0433
RT-NMB0433-rev	TGAGTCAGTACCGACGCAGGTA	
RT-NMB0434-for	AGCTCGACGGCGTAACGT	RT-PCR of NMB0434
RT-NMB0434-rev	CGTCGGCTGGATCAAGAAAT	
RT-NMB1799-for	GCCTGCCAATACGCACAAG	RT-PCR of *metK*
RT-NMB1799-rev	GCGCAAGACCCAAAAGCA	

		439543-439560
RT-NMB0428-rev*	CGTCATATCGTCCCAATCAATATC	439612-439589
RT-NMB0430-for*	GCCGTGAAAGAATCGAATCC	440460-440479
RT-NMB0430-rev*	TGGCCAATCGTGCAAAATAA	440526-440507
RT-NMB0431-for	GCCATGCACGTTTCACTGAT	441937-441956
RT-NMB0431-rev	CGCGGGCGGTAAAGGTA	442003-441987
RT-NMB0432-for	TGCAATCTTGGTTCGCTATCG	443247-443267

RT-NMB0432-rev	CCATCGTTGCCGCAATC	443316-443300
RT-NMB0433-for	CGCCCGTCGTCCAAGTC	444114-444130
NMB0433b-for*	AGGCTTCGAGCGTATCCAC	445870-445888
RT-NMB0433-rev	TGAGTCAGTACCGACGCAGGTA	444175-444154
RT-NMB0434-for	AGCTCGACGGCGTAACGT	447445-447462
RT-NMB0434-rev	CGTCGGCTGGATCAAGAAAT	447507-447488
RT-NMB0435-for*	CGCATGATTATTGCCCACTTAG	449150-449171
RT-NMB0435-rev*	GACGGATTTGCCGTTTTTGA	449215-449196
RT-NMB0436-for*	GAAGACGGCGAACCATTGA	450552-450570
RT-NMB0436-rev*	TCGACGGCGTGTTCCAA	450613-450597

Dataset S1. Pairwise similarity between taxa (defined by 97 % identity) assessed using Pearson's Product Moment Correlation based on the data from Nasidze et al. (2009).

Dataset S2. Pairwise similarity between taxa (defined by 97 % identity) assessed using Pearson's Product Moment Correlation based on the data from Stahringer et al. (2012).

Dataset S3. Pairwise similarity between taxa (defined by 97 % identity) assessed using Pearson's Product Moment Correlation based on the data from Charlson et al. (2010).

Dataset S4. Pairwise similarity between taxa (defined by 97 % identity) assessed using Pearson's Product Moment Correlation based on the data from unpublished study by the Human Microbiome Project, available on the Sequence Read Archive under accession number SRX055077.

Dataset S5. Pairwise similarity between taxa (defined by 99 % identity) assessed using Pearson's Product Moment Correlation based on the data from Charlson et al. (2010).

Dataset S6. Pairwise similarity between taxa (defined by 99 % identity) assessed using Pearson's Product Moment Correlation based on the data from unpublished study by the Human Microbiome Project, available on the Sequence Read Archive under accession number SRX055077.
